# Characterization of Distinct Microbiota Associated with Scalp Dermatitis in Patients with Atopic Dermatitis

**DOI:** 10.3390/jcm11061735

**Published:** 2022-03-21

**Authors:** Yu Ri Woo, Minah Cho, Yujin Han, Se Hoon Lee, Sang Hyun Cho, Jeong Deuk Lee, Hei Sung Kim

**Affiliations:** Department of Dermatology, Incheon St. Mary’s Hospital, The Catholic University of Korea, Seoul 06591, Korea; w1206@naver.com (Y.R.W.); macho_maria@naver.com (M.C.); thyme3700@gmail.com (Y.H.); leesehoon92@gmail.com (S.H.L.); drchos@yahoo.co.kr (S.H.C.); leejd@catholic.ac.kr (J.D.L.)

**Keywords:** atopic dermatitis, bacteria, fungus, microbiota, scalp

## Abstract

Recent studies have focused on the role of skin microbiota in the pathogenesis of atopic dermatitis (AD). Among the various clinical phenotypes of AD, scalp dermatitis is a commonly observed clinical feature of AD. However, little is known about the pathogenesis of scalp dermatitis in AD. Hence, the aim of this study was to identify the distinct microbiota associated with scalp dermatitis in patients with AD. Using scalp swab samples from 10 patients with AD and 10 healthy controls, this study characterized the scalp microbiota in patients with AD via V3–V4 regions of the 16S rRNA gene sequencing for bacterial identification, and ITS2 gene sequencing for fungal identification. Among bacterial genera, *Staphylococcus* was the most abundant in AD than in healthy controls, whereas *Cutibacterium* was the most abundant species in the healthy controls. The most predominant scalp fungal microbiota was *Malassezia* both in AD and healthy controls, while a higher diversity of non-Malassezia fungi was observed in AD than in healthy controls. The study findings indicate the dysbiosis of scalp microbiota in AD and highlight the potential biomarker role of specific microbiota in AD on the scalp dermatitis.

## 1. Introduction

Atopic dermatitis (AD) is a common chronic inflammatory skin disorder with an increasing prevalence worldwide. The clinical presentation of AD varies according to age, ethnicity, genetic predisposition, and environmental risk factors. In general, the major clinical features of AD based on Hanifin and Rajka diagnostic criteria include pruritus, typical morphology and distribution, chronic and relapsing dermatitis, personal or family history of atopy, and early age of onset [1]. In addition to these features, various minor clinical features of AD are suggested due to the heterogeneity of AD.

Among minor clinical features of AD, scalp involvement of AD is commonly observed in various studies [2,3,4]. The study by Shi et al. [4] suggested that scalp dermatitis was observed in 49.7% of patients with AD. The scalp dermatitis associated with AD is also observed as diffuse scaling of the scalp [5], or dandruff [2] in various studies. However, most of the studies of adult AD to date have focused on the flexural area of the skin, and little is known about the clinical features and pathogenesis of scalp dermatitis in patients with AD.

Recently, the role of skin microbiota was implicated in AD pathogenesis. In patients with AD, the dysbiosis of skin microbiota is predominantly associated with *Staphylococcus aureus* [6]. The colonization of *S. aureus* is also positively correlated with disease severity [7]. However, most of the studies of the microbiome in AD involved skin lesions including antecubital and popliteal fossae, cheek, groin, and ear flexure [8,9,10]. As the skin commensals differ according to the body sites, identification of scalp microbiota in patients with AD provides insight into the pathogenesis of scalp dermatitis. Indeed, the scalp is characterized by a high density of pilosebaceous units and sweat glands, which increases the sebum content. As sebum is a pivotal source of bacterial and fungal growth, the characterization of scalp microbiota in patients with AD requires further elucidation.

Therefore, this study aims to analyze the distinct microbiota associated with scalp dermatitis in patients with AD. To identify the distinct scalp microbiota in AD, bacterial and fungal taxa in patients with AD were analyzed and their composition was compared with healthy controls. In addition, the clinical characteristics of AD were matched with the microbial composition to determine the characteristic biomarkers of scalp dermatitis.

## 2. Methods

### 2.1. Study Participants

Patients who were diagnosed with AD by a board-certified dermatologist based on Korean diagnostic criteria of AD [11] from July 2021 to August 2021 at the Department of Dermatology, Incheon St. Mary’s Hospital, Korea, were enrolled in this study. AD patients with scalp dermatitis alone were included in this study. The overall clinical severity of AD was assessed using Eczema Area and Severity Index (EASI) scores. In the absence of a specific tool for examining the severity of scalp dermatitis in AD to date, we have used the validated Investigator Global Assessment scale for Atopic dermatitis (vIGA-AD^TM^) to evaluate the severity of scalp dermatitis in patients with AD [12].

For comparison, the study included healthy individuals without a previous history or concomitant AD, seborrheic dermatitis (SD), or any specific skin lesions on the scalp. The exclusion criteria were patients younger than 18 years, a previous history of systemic or topical antibiotic and/or antifungal therapies within 4 weeks of study, history of skin cancer, and a recent use of anti-seborrheic or anti-dandruff shampoos within 4 weeks. All participants who provided written informed consent prior to the survey were enrolled. This study was approved by the ethics committee of Incheon St. Mary’s Hospital and was conducted according to the principles of the Declaration of Helsinki (OC21TASI0068).

Skin samples were collected by the same investigator (Y.R.W) from the vertex of the scalp (a single sampling covering a 4 cm^2^ area) using sterile cotton swabs (4N6 FLOQSwabs^®^, Copan Diagnostic Inc., Brescia, Italy).

### 2.2. DNA Extraction

DNA was extracted from skin samples using a DNeasy PowerSoil Pro Kit^TM^ (QIAGEN, Valencia, CA, USA) according to the manufacturers’ protocol. The DNA levels were measured with PicoGreen and the DNA quality was analyzed using NanoDrop.

### 2.3. Polymerase Chain Reaction Amplification and Sequencing

After extraction of DNA, the V3–V4 hypervariable regions of the 16S rRNA gene were amplified with 16S V3–V4 upstream (5′-TCGTCGGCAGCGTCAGATGTGTATAAGAGACAGCCTACGGGNGGCWGCAG-3′) and downstream (5′-GTCTCGTGGGCTCGGAGATGTGTATAAGAGACAGGACTACHVGGGTATCTAATCC-3′) primers to identify the fungal taxa. According to the manufacturer’s instructions, the input gDNA was amplified with 16S V3–V4 primers, followed by ITS primers. A subsequent limited cycle-amplification step was performed for additional multiplexing indices and Illumina sequencing adapters. The final products were normalized and pooled using PicoGreen, and the size of the libraries was identified using the LabChip GX HT DNA High Sensitivity Kit (PerkinElmer, Waltham, MA, USA), followed by sequencing using the MiSeq™ platform (Illumina, San Diego, CA, USA) with 301 bp paired-end reads.

### 2.4. Sequence Processing and Diversity Estimation

The prepared library was sequenced with the Illumina MiSeq^TM^ platform. Following completion of sequencing, the MiSeq raw data were classified in the samples using an Index sequence, and a FASTQ file for each sample was generated. Using fastp, the adapter sequences were removed and error correction was performed [13]. The paired-end reads were merged using FLASH v1.2.11 [14]. The raw reads containing sequence errors (i.e., merged sequences under 400 bp, over 500 bp, raw reads with ambiguous base cells, or chimeric sequences) were removed by CD-HIT OTU [15]. The remaining reads were de novo clustered into operational taxonomic units (OTUs) with sequences showing a 97% similarity cutoff.

The representative sequences of each OTU in the fungal ITS2 sequences were taxonomically assigned using the reference database (UNITE Fungi2 99% clustering v8.2) and BLAST v2.9.0+ (https://blast.ncbi.nlm.nih.gov/Blast.cgi, accessed on 25 December 2021) [16]. The taxonomic assignment of OTUs in the bacterial 16S rRNA sequences from each sample was performed using the reference database of NCBI 16S Microbial database, and BLAST+v2.9.0, http://blast.ncbi.nlm.nih.gov/Blast.cgi, accessed on 25 December 2021) [16].

To improve the accuracy of taxonomic assignments, a top BLAST hit with alignment spanning < 85% of the original query sequence and a top BLAST hit with a percent identity less than 85% were not assigned. The observed relative abundances were estimated by dividing the observed number of 16S and ITS2 rRNA amplicon reads by the total number of reads per sample. The phylogenetic tree was constructed using QIIME’s script make_phylogeny.py with the FastTree algorithm. The α diversity of each sample was measured using Shannon and Chao1 indices using QIIME v1.9 [16]. Unweighted and weighted UniFrac distances were calculated using the beta_diveristy.py script from QIIME (v1.9). The principal coordinate analysis (PCoA) was conducted to provide an intuitive visualization of the data structure and look at differences between the samples using QIIME v1.9.

### 2.5. Statistical Analysis

The comparison of α diversity and the mean relative abundance of bacterial and fungal taxa between two groups was conducted with Wilcoxon rank sum tests using R statistical software, version 3.6.2. The Benjamini–Hochberg correction was applied for multiple testing correction. The analysis of similarities (ANOSIM) with 999 permutations was conducted to compare the statistical differences between two groups in PCoA by QIIME v1.9. The Linear discriminant Effect Size (LEfSe) was performed to analyze the OTUs with differential relative abundance between AD and healthy control (HC) groups. The degree of difference was expressed as a linear discriminant analysis (LDA) score with a cutoff value of ≥2.00. For all statistical analyses, a two-sided *p* value of less than 0.05 was statistically significant.

## 3. Results

### 3.1. Demographic Characteristics and Results of Sample Clustering Analysis

We analyzed the bacterial and fungal microbiota from 20 different subjects. Participants’ demographic characteristics are summarized in Table 1. This study included 10 patients with AD diagnosed with scalp dermatitis and 10 healthy controls. The mean age of patients with AD of the scalp dermatitis was 33.2 years with a standard deviation (SD) of 11.80, and that of the healthy controls was 37.7 ± 10.36 (mean ± SD) years. Based on the clinical severity of AD according to the EASI, six patients were diagnosed with mild AD, while four patients were diagnosed with moderate-to-severe AD. No statistically significant difference was found between the two groups regarding age and sex.

We obtained a dataset consisting of 20 samples sequenced as a total high-quality read count of 710,904, with a median of 32,767 for each sample derived from bacterial 16S rDNA V3–V4 sequences (Table S1). A total of 341 OTUs were identified for bacterial microbiota at a cutoff value of 97% similarity at species level. For fungal ITS, a dataset consists of 20 samples sequenced as a total high-quality read count of 1,645,279 with a median of 82,438 for each sample. A total of 121 OTUs were identified for fungal microbiota in this analysis.

### 3.2. Diversity Analysis

**Figure 1:**
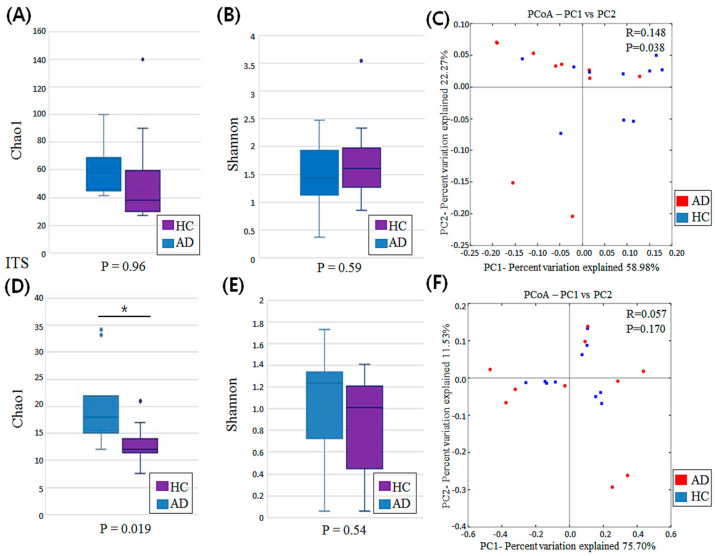
Comparison of bacterial and fungal diversity associated with scalp dermatitis in atopic dermatitis (AD) and healthy control (HC) groups. (**A**,**B**) Alpha-diversity of bacterial microbiota among AD and HC groups. (**C**) A two-dimensional plot of principal coordinate analysis (PCoA) based on weighted UniFrac distances for bacterial microbiota between AD and HC groups, and analysis of similarities (ANOSIM) for evaluating beta diversity between two groups. (**D**,**E**) Alpha diversity of fungal microbiota in AD and HC groups. (**F**) A two-dimensional plot of PCoA based on weighted UniFrac distances for fungal microbiota between AD and HC groups, and results of ANOSIM for beta diversity between two groups. * indicates a significant *p*-value of less than 0.05.

To identify the α diversity of each sample, Shannon index and Chao1 were used. The Chao1 index (richness of each sample) for bacterial population was slightly higher in the AD group than in the HC group, but there was no statistically significant difference between the groups (Figure 1A). The values of the Shannon index (evenness and abundance) for bacterial population were slightly lower in the AD group than in healthy controls; however, this difference was not statistically significant (Figure 1B). The β diversity based on ANOSIM was conducted to identify the degree of difference in the bacterial population, including the PCoA based on weighted UniFrac distances. The three-dimensional PCoA of weighted UniFrac distances showed a mild clustering among bacterial microbiota between AD and HC groups by ANOSIM (R = 0.148; *p* = 0.038; Figure 1C).

The Chao1 index for fungal population was higher in the AD group than in the HC group (*p* = 0.019; Figure 1D). No significant differences were found in Shannon indices (Figure 1E). The PCoA based on weighted UniFrac distances was also used to compare the β diversity of each sample for fungal microbiota. Three-dimensional PCoA plots revealed no specific clustering in fungal microbiota between AD and HC groups by ANOSIM (R = 0.057; *p* = 0.17; Figure 1F).

### 3.3. Composition of Bacterial Microbiota in the Scalp Based on Taxonomy

**Figure 2:**
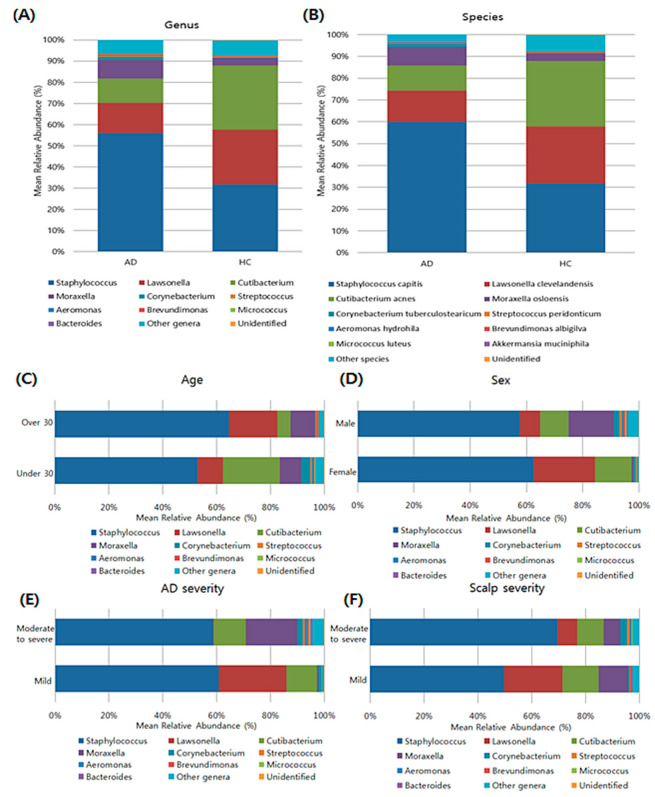
Composition of abundant bacterial genera (**A**) and species (**B**) in AD and HC groups, and composition based on clinical characteristics of AD group (**C**–**F**).

A total of 12 bacterial phyla were identified, and most sequences were assigned to *Actinobacteria* (48.68%), *Firmicutes* (42.25%), and *Proteobacteria* (8.28%). At the genus levels, a total of 195 genera were identified. Among them, *Staphylococcus, Lawsonella, Cutibacterium, Moraxella*, *Corynebacterium, Streptococcus, Aeromonas, Brevundimonas, Micrococcus,* and *Bacteroides* were the 10 most abundant bacterial genera in the AD group (Figure 2). At the species level, *Staphylococcus capitis* was the most abundant in the scalp samples of the AD group, followed by *Lawsonella clevelandensis, Cutibacterium acnes*, and *Moraxella osloensis*. However, when we divided the AD samples into two groups according to their age, gender, and scalp severity, there was no statistical difference in the mean relative abundance of bacterial genera according to their clinical characteristics. The patients with mild AD showed to have the increased relative abundance of *Lawsonella* than patients with moderate to severe AD (false discovery rate (FDR)-adjusted *p* = 0.02).

### 3.4. Composition of the Fungal Microbiota in the Scalp at the Taxonomic Assignment

**Figure 3:**
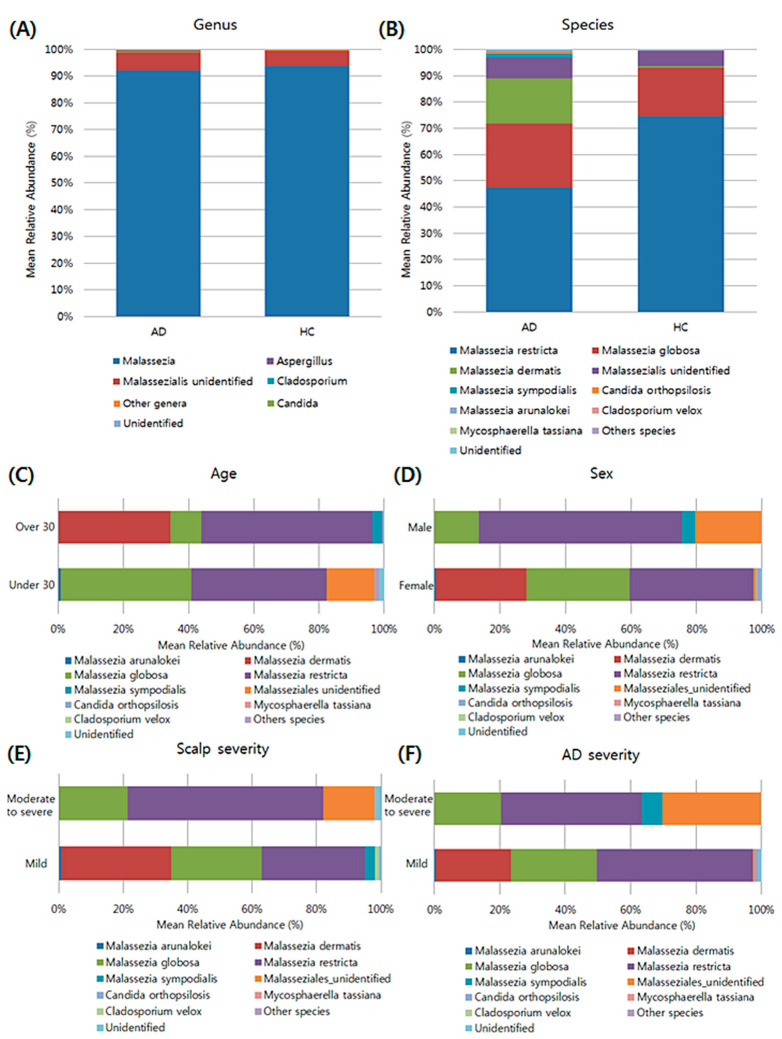
Composition of the abundant fungal genera (**A**) species (**B**) in AD and HC groups, and the composition based on clinical characteristics of AD group (**C**–**F**).

Most of the sequences in the four identified fungal phyla were assigned to *Basidiomycota* (99.20%). A total of 71 genera were identified and most of the genera in the AD and HC groups were *Malassezia,* followed by unidentified genera belonging to family *Malasseziaceae* (Figure 3). At the species level, *Malassezia restricta* was the most abundant in the scalp samples of AD and HC groups, followed by *M. globosa*. When we divided the AD samples into two groups according to their clinical characteristics, there was no statistically significant difference in the mean relative abundance of their fungal species according to patients’ age, gender, AD severity, and scalp severity. Further, the mean ratio of *M. restricta* to *M. globosa* was increased in the AD group than in the HC group; however, no statistically significant difference was observed.

### 3.5. Taxonomic Biomarkers of AD and HC Groups

Based on the result of taxonomic assignment, we further conducted Linear Discriminant Analysis Effect Size (LEfSE) analysis to identify specific bacterial and fungal taxa that showed a significant difference between AD and HC groups, and to examine how much difference is observed between two groups using a log scale.

**Figure 4:**
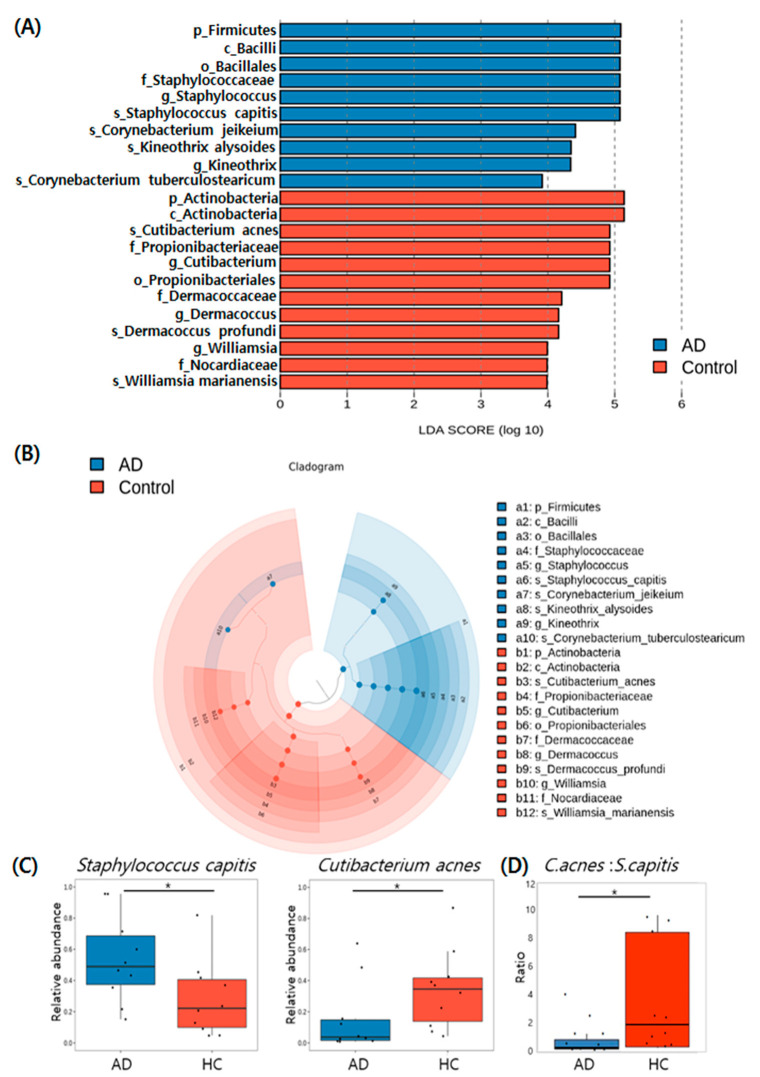
Results of Linear Discriminant Analysis Effect Size (LEfSe), analysis of scalp bacterial microbiota in AD and HC groups. (**A**) Histogram of LDA scores depicting differential abundance of bacterial microbiota between AD and HC groups. (**B**) Cladogram of LEfSe of the skin microbiome based on 16S rRNA sequencing. (**C**) Box plots representing statistically significant dominant bacterial species with an average relative abundance greater than 0.1% between AD and HC groups. (**D**) Box plots representing the ratio of *Cutibacterium acnes* to *Staphylococcus capitis* in the AD and HC groups (*p* = 0.019). Asterisk (*) indicates a significant difference based on *p*-values less than 0.05.

The LEfSe analysis revealed that 22 bacterial clades varied statistically between the two groups. Among the identified genera, two bacterial genera (*Staphylococcus* and *Kineothrix*) were abundant in the AD group, while three genera (*Cutibacterium, Dermacoccus, and Williamsia*) were enriched in the HC group (Figure 4A). The LEfSe cladogram demonstrates the differential abundance of taxa in scalp microbiota between the two groups (Figure 4B). The bacterial species that showed statistically significant abundance in the AD group compared to the HC group were *Staphylococcus capitis* (FDR-adjusted *p* = 0.04), *Cornynebacterium jeikeium* (FDR-adjusted *p* = 0.04)*, and Kineothrix alysoides* (FDR-adjusted *p* = 0.04). Statistically significant levels of abundant bacterial species were higher in the HC group than in the AD group and were identified as *Cutibacterium acnes* (FDR-adjusted *p* = 0.04)*, Dermacoccus profundi* (FDR-adjusted *p* = 0.04), and *Williamsia marianensis* (FDR-adjusted *p* = 0.04). Analysis of dominant species with an average relative abundance greater than 0.1% revealed that *S. capitis* was more abundant in the AD group than in the HC group, while *Cutibacterium acnes* was the most abundant species in the HC group than in the AD group (Figure 4C). Further, the mean ratio of *Cutibacterium acnes* to *Staphylococcus capitis* decreased in the AD group compared to the HC group (*p* = 0.019; Figure 4D).

**Figure 5:**
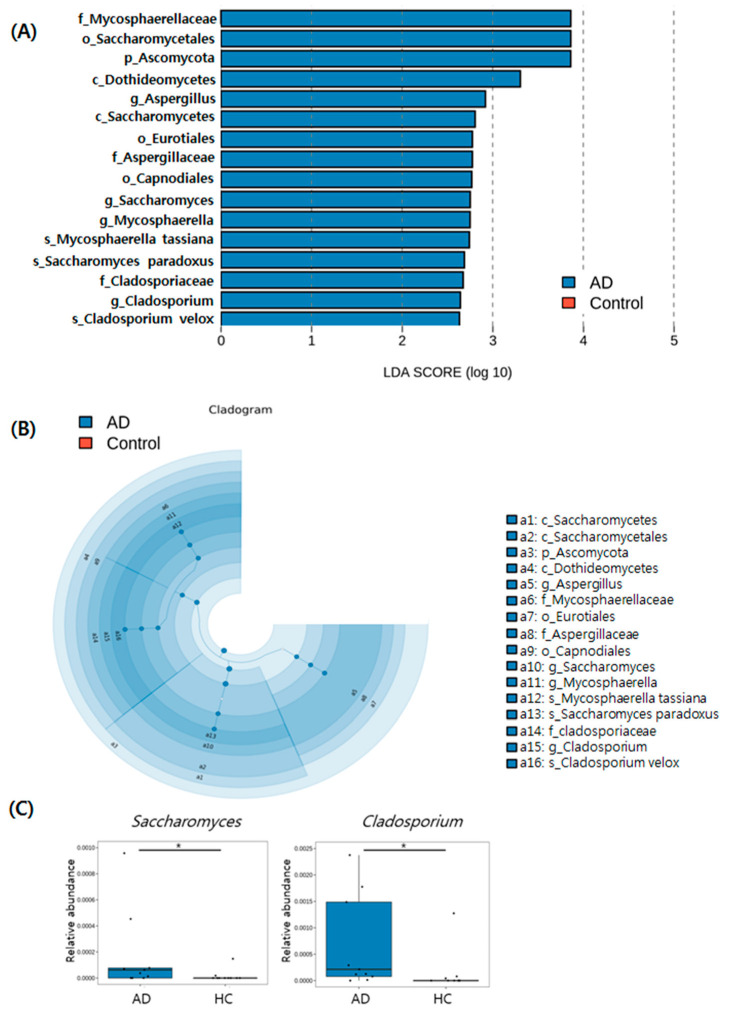
Results of Linear Discriminant Analysis Effect Size (LEfSe), analysis of scalp fungal microbiota in AD and HC groups. (**A**) Histogram of LDA scores representing differential abundance of fungal microbiota between AD and HC groups. (**B**) Cladogram of LEfSe of skin microbiome based on ITS sequencing analysis. (**C**) Box plots representing statistically significant dominant fungal genera based on the relative abundance of specific genera between AD and HC groups. Asterisk (*) indicates a significant difference based on *p*-values less than 0.05.

The LEfSe analysis identified 16 fungal clades that varied statistically significantly between the two groups (Figure 5A). The LEfSe cladogram demonstrates differential abundance of fungal taxa in scalp between the two groups (Figure 5B). Significantly higher levels of *Saccharomyces* (FDR-adjusted *p* = 0.03) and *Cladosporium* (FDR-adjusted *p* = 0.01) were found in AD in comparison to the HC group (Figure 5C).

## 4. Discussion

This study provides a comprehensive sequence analysis of scalp microbiota in a patient cohort diagnosed with AD. To date, *Malassezia* has been implicated as the major cause of scalp dermatitis in patients with AD based on findings of previous culture-based studies [17,18]. In line with the previous findings, we found that *Malassezia* is the most dominant fungal genus in the AD scalp. In addition to the fungal microbiota, we found differences in bacterial compositions between patients with AD and healthy individuals. Of note, we found that *Staphylococcus capitis* was the most abundant in AD compared to the HC, whereas *Cutibacterium acne* was the most abundant species in the HC compared to the AD group in this study. *Staphylococcus* is a major bacterial taxon affecting skin in both AD and HC. The results presented here are consistent with previous findings [19,20]. *Staphylococcus capitis* was enriched more in the scalp of AD patients with scalp dermatitis than those in the HC group. The overabundance of *Staphylococcus capitis* was also reported in the study of dandruff-affected scalp by Grimshaw et al. [19]. In addition to *Staphylococcus aureus*, clusters enriched by *S. capitis* and *S. epidermidis* were also found [20,21], suggesting the possible existence of diverse Staphylococcal communities in AD skin. *Staphylococcus* is well known to affect the clinical course of AD by its capability to activate virulence factors such as exogenous proteases, staphylococcal superantigens, staphylococcal α-toxin and phenol soluble modulins [22]. These virulence factors exert a negative effect on patients with AD by disrupting epithelial barrier integrity and the immune system.

Interestingly, we found a greater abundance of *Cutibacterium acnes* in healthy scalp than in AD, and the ratio of *Cutibacterium acnes* to *Staphylococcus capitis* was higher in HC than in AD. The study findings are similar to those of a previous scalp microbiome analysis, suggesting the abundance of *Cutibacterium* in healthy scalp than in seborrheic dermatitis [23,24]. *Cutibacterium* is dominant in sebaceous areas and suppresses the growth of *Staphylococcus* via fermentation of glycerol [25,26]. Moreover, the colonization of *Cutibacterium* depends on various host factors, including humidity, acidity, temperature, and lipid composition [27]. Further studies are needed to determine whether the decrease in the mean relative abundance of *Cutibacterium* in AD is due to physiological scalp condition or the antagonism between *Staphylococcus* and *Cutibacterium*. In contrast to *Cutibacterium*, *Corynebacterium* spp. was enriched in the scalp of AD compared to the HC. A recent study investigating AD also suggested that bacteria in the forehead include predominantly *Staphylococcus,* followed by *Corynebacterium* [28]. In a mouse model of AD, the increased abundance of *Corynebacterium* along with *Staphylococcus* suggested that *Staphylococcus* was primarily involved in eczema, whereas *Corynebacterium* induced Th2 cell responses [29]. Therefore, we can assume that the overabundance of *Corynebacterium* enhanced inflammation of AD skin along with *Staphylococcus*.

We found that patients with moderate to severe AD showed the tendency of increased relative abundance of Moraxella than patients with mild AD, although this difference was not statistically significant. *Moraxella* is a Gram-negative coccobacillus and known as a skin commensal. However, it can cause opportunistic infections in some immunocompromised individuals. Increased abundance of *Moraxella osloensis* is reported in some patients with AD and asthma [30,31]. As many patients with severe AD are prescribed various immunosuppressants, further research is needed on the functional role of *Moraxella* in AD.

Kong et al. [32] suggested that use of the V1–V3 hypervariable region of 16S rRNA was more accurate in identifying common skin bacteria, and the use of the V4 hypervariable region of the 16S rRNA gene might lead to an underestimation of some genera. However, due to the longer amplicon size of the V1–V3 region compared to V3–V4, the merge rate of the paired-end reads is higher in the V3–V4 region than in the V1–V3 region. [33]. Therefore, we used the V3–V4 hypervariable region of 16S rRNA to identify the skin bacterial microbiota in the present study.

The fungal microbiota in the scalp of patients with AD and HC was dominated by *Malassezia restricta* followed by *Malassezia globosa*, which is consistent with the study of Oh et al. [34]. To date, the role of *Malassezia* in AD pathogenesis has been demonstrated in various studies. Indeed, treatment with antifungal agents decreased the clinical severity of AD [35,36]. However, there was no significant difference in the mean relative abundance of *Malassezia*, the most abundant fugal taxa in the scalp, between the two groups in this study. The study findings are in accordance with the study by Soares et al. [37]. The findings showed that the composition of the most dominant fungal species in the scalp, *Malassezia*, was similar between healthy individuals and AD patients. Healthy individuals are not always sensitized with *Malassezia* species, whereas a higher proportion of patients with AD are sensitized with *Malassezia* species [38], suggesting fungal interaction with the host’s skin immune system. To date, various *Malassezia* antigens associated with AD have been found including Mala S1-9, MGL 1304, Mala s 8, Mala s 13, and Mala r 8 [39,40,41,42]. We suggest that the interaction between various *Malassezia* antigens and the skin’s immune system further promoted inflammation in AD.

In addition, we found a greater increase in the mean relative abundance of *Malassezia dermatitis* in AD than in the HC groups, which was in line with a previous study [43]. However, the difference was not statistically significant, and a further study is needed to corroborate this association. Further, the higher abundance of *M. dermatis* in patients with AD aged above 30 years than in those under 30 years suggests the need for further large-scale studies to establish their clinical relevance.

Of note, we found differences between AD and healthy individuals in the abundance of fungal microbiota in the scalp, based on LEfSe analysis. *Aspergillus, Saccharomyces, Mycosphaerella,* and *Cladosporium* were identified as statistically significant fungal taxa in AD and HC groups via LEfSe analysis. Although *Malassezia* species were the most abundant fungi in the two groups, a further fungal diversity of non-Malassezia species was observed in AD than in HC. The study findings are consistent with a previous systematic review of AD, reporting additional fungal diversity of non-Malassezia species in AD than in HC [43]. The significance of *Aspergillus, Cladosporium, and Saccharomyces* in allergic disease is well established. A recent study by Celakovska et al. [44] found a positive correlation between the sensitization of molecular components of *Saccharomyces, Aspergillus,* and *Cladosporium* with the clinical severity of AD. *Mycosphaerella* is a plant pathogen and is transmitted by aerial contamination. *Mycosphaerella* was also enriched in the scalp of patients with seborrheic dermatitis [45], and in patients with asthma [46]. Therefore, a further study is needed to determine whether the enrichment of *Mycosphaerella* is due to scalp inflammation or atopy.

This study is limited by the small sample size. In addition, we did not sequence for the negative controls. Although a negative control sample was not prepared in this study, in order to increase the accuracy of community diversity comparison analysis between AD and control groups, everything from the sampling method to the batch of sequencing equipment was carried out under the same conditions. Usually, contamination issues are confirmed in amplicon metagenome studies when different DNA prep kits are used, PCR conditions are different, or batches are different. These potentials have been minimized throughout this study. Our study did not analyze the correlation between the bacteriome and mycobiome data, suggesting the need for further large-scale studies to identify the interaction between microbiota including bacterial and fungal taxa residing in the complex host environment.

In conclusion, our findings add to the evidence suggesting that dysbiosis of scalp microbiota in AD might play a pivotal role in scalp dermatitis of AD. Previously, scalp dermatitis in AD was mainly treated with antifungal agents targeting *Malassezia*. The study findings establish that not only *Malassezia*, but also changes in bacterial and non-Malassezial fungal microbiota occur in scalp dermatitis of AD. Therefore, the therapeutic targets in AD of the scalp should focus on both the bacterial and fungal microbiota based on the findings of this study. A further study is also warranted to confirm the role of specific microorganisms in AD pathogenesis, and the resulting defects associated with skin barrier, skin immune system, and inflammation.

## Figures and Tables

**Table 1:** Demographic characteristics of the study population.

	Atopic Dermatitis (*n* = 10)	Healthy Control (*n* = 10)
Age	33.2 ± 11.80 (19–53)	37.7 ± 10.36 (28–63)
Gender		
Male	5 (50%)	5 (50%)
Female	5 (50%)	5 (50%)
EASI		N/A
Mild (<6)	6 (60%)	
Moderate to severe (≥6)	4 (40%)	
vIGA-AD for scalp dermatitis		N/A
Mild	5 (50%)	
Moderate to severe	5 (50%)	

Data were presented as number (%) or mean ± standard deviation. Abbreviation: N/A, not accessible.

## Data Availability

The data that support the findings of this study are available on request from the corresponding author. However, the data are not publicly available due to the ethical restriction.

## Supplementary Materials

The following supporting information can be downloaded at: https://www.mdpi.com/article/10.3390/jcm11061735/s1, Table S1. Summary for total read count of sequence reads.
