# Supplementary material for: Characterization of Distinct Microbiota Associated with Scalp Dermatitis in Patients with Atopic Dermatitis
Source: J Clin Med. 2022 Mar 21;11(6):1735. doi: 10.3390/jcm11061735 (PMC8949928; doi:10.3390/jcm11061735)
Supplement: Supplementary file 1 [file jcm-11-01735-s001.zip › jcm-1549948-supplementary.pdf]

Table S1. Summary for total read count of sequence reads.

Group/Sample No.	Raw Read Count	Sequence Read Count Aligned with 16S	Raw Read Count	Sequence Read Count Aligned with ITS
AD/1	177,454	34,966	190,052	79,102
AD/2	164,248	26,628	206,148	74,074
AD/3	174,332	24,859	200,694	79,675
AD/4	172,536	50,979	192,684	70,398
AD/5	184,038	33,435	187,940	69,945
AD/6	201,800	52,833	213,256	80,432
AD/7	207,502	74,412	214,662	80,070
AD/8	159,074	29,288	248,738	103,180
AD/9	173,794	40,660	202,704	83,818
AD/10	247,762	44,944	233,566	99,933
HC/1	183,616	30,992	205,880	87,902
HC/2	180,242	32,670	192,514	84,235
HC/3	156,024	32,865	219,392	86,844
HC/4	159,120	23,820	211,002	85,303
HC/5	192,056	19,775	195,186	84,823
HC/6	193,298	34,821	160,252	50,251
HC/7	158,172	47,980	228,002	96,715
HC/8	169,184	29,357	198,740	81,057
HC/9	155,000	13,825	229,046	91,723
HC/10	205,214	31,795	205,296	75,799
